# A fast electrochromic polymer based on TEMPO substituted polytriphenylamine

**DOI:** 10.1038/srep30068

**Published:** 2016-07-22

**Authors:** Lvlv Ji, Yuyu Dai, Shuanma Yan, Xiaojing Lv, Chang Su, Lihuan Xu, Yaokang Lv, Mi Ouyang, Zuofeng Chen, Cheng Zhang

**Affiliations:** 1 State Key Laboratory Breeding Base of Green Chemistry-Synthesis Technology, College of Chemical Engineer, Zhejiang University of Technology, Hangzhou 310014, China; 2 Department of Chemistry, Tongji University, Shanghai 200092, China; 3 College of Chemical Engineering, Shenyang University of Chemical Technology, Shenyang 110142, China; 4 Department of Chemistry, Tsinghua University, Beijing 100084, China

## Abstract

A novel strategy to obtain rapid electrochromic switching response by introducing 2,2,6,6-tetramethyl-1-piperidinyloxy (TEMPO) moiety into polytriphenylamine backbone has been developed. The electrochromic properties of the integrated polymer film are investigated and a possible mechanism is proposed with TEMPO as a counterion-reservoir group to rapidly balance the charges during electrochromic switching, which leads to significantly improved electrochromism performance.

Electrochromism (EC) is a phenomenon showed by chemical materials of optical change by a reversible electrochemical process^1^,^2^,^3^. This interesting property leads to the development of several technological applications, such as smart windows^4^, rear-view mirrors and visors^5^, electronic papers and EC displays^6^. Initial researches on EC materials mainly focused on inorganic metal oxides such as WO_3_^7^, transition metal complexes such as Prussian blue^8^ and low molecular weight organic materials such as viologen^9^. Recently, conducting polymers have attracted much attention because of their superior EC properties, such as easy processability, high optical contrast ratio, fast switching response and multi-color tenability^10^,^11^,^12^.

Triphenylamine (TPA) and its derivatives are well-known for their photo and electroactive properties, which have been widely used as the hole-transport layer in electroluminescent diodes^13^,^14^. Moreover, these materials also exhibit interesting electrochromic properties^15^. For unsubstituted TPA, it could undergo rapid coupling deprotonation to form tetraphenylbenzidine during the anodic oxidation, which would hinder its further electropolymerization^16^. To solve this problem, electron-rich groups were usually grafted to the *para*-position of TPA^17^. A variety of TPA-based electrochromic polymers have been reported in literature^18^,^19^,^20^,^21^,^22^.

2,2,6,6-Tetramethyl-1-piperidinyloxy (TEMPO) is well-known as a stable nitroxyl radical with potential applications in pharmaceutics, catalysis, non-linear optical materials and cathode materials for rechargeable lithium batteries^23^,^24^,^25^. As a transparent organic radical provider that undergoes a stable and reversible redox reaction in organic solvents, TEMPO usually does not exhibit electrochromic behaviors. Even though, there are a few studies using TEMPO or TEMPO-based polymers as electroactive materials for the counter-electrode in electrochromic devices^26^,^27^,^28^. For example, Takahashi *et al.* prepared an electrochromic cell using a TEMPO-based polymer film as the counter-electrode, allowing the device working at a remarkably low cell voltage^29^. Hu *et al.* employed TEMPO as a stable radical provider at the counter-electrode of an EC device, achieving a superior EC performance^30^.

However, to our best knowledge, there is no report describing the application of TEMPO as a component of EC material. As TEMPO shows many advantages, especially for its excellent electrochemical performance, we speculate that TEMPO may serve as a counterion-reservoir group in EC materials rather than just as electroactive materials for counter-electrode in EC devices. In this work, we synthesized a TEMPO-containing triphenylamine derivative, 4-carboxy-*N,N*-diphenylaniline-2,2,6,6-tetramethylpiperidin-1-yloxy (TPAT)^31^, and electropolymerized it on an ITO (Sn(IV)-doped In_2_O_3_) substrate (PTPAT). The electrochromic and spectroelectrochemical properties of PTPAT were studied which exhibits significantly improved EC performance. The possible electrochromic mechanism related with the important role played by TEMPO moieties is proposed.

## Experimental

### Materials

Diphenylamine (98%), 4-fluorobenzonitrile (99%), 4-hydroxy-2,2,6,6-tetramethylpiperidine 1-oxyl free radical (99%), lithium perchlorate (LiClO_4_, 99%), and chromatography grade acetonitrile (CH_3_CN) were purchased from Energy Chemical Reagent Co. Sodium hydride (60%) was purchased from Aladdin. All other reagents were of analytical grade and used as received without further purification. Indium tin oxide (ITO) glass substrates (CSG HOLDING Co., Ltd, *Rs *≤ 10 Ω^−1^) were used after ultrasonic washing in distilled water, ethanol, toluene and acetone solutions, respectively.

### Instrumentation

FT−IR spectra were obtained on a Nicolet 6700 spectrometer (Thermo Fisher Nicolet, USA) with KBr pellets. Scanning electron microscopy (SEM) measurements were performed using a Hitachi S-4800 scanning electron microscope (Hitachi, Japan). The electrochemical properties and long-term stability test were measured in a three-electrode cell (ITO/glass electrode as the working electrode, platinum wire electrode as the counter electrode and Ag/AgCl electrode as the reference electrode) with a CHI 660E electrochemical workstation in a 0.1 M LiClO_4_/CH_3_CN solution with *iR* compensation. UV-Vis spectra, optical contrast and switching time measurements were carried out on a Shimadzu UV-1800 UV-Vis spectrophotometer (Shimadzu, Japan). The contact angle measurement was recorded on a Datahpysics OCA30 (Germany).

### Synthesis of the TPAT monomer

*Synthesis of compound **1*** (*4-cyano-N,N-diphenylaniline*) Diphenylamine (5.1 g), sodium hydride (1.5 g) and *N,N*-dimethylformamide (DMF, 50 mL) were firstly mixed in a pre-dried flask, and then 4-fluorobenzonitrile (4.5 g) was added. The mixture was performed under a N_2_ atmosphere for 12 h at 110 °C. After cooling, the resulting solution was extracted with CH_2_Cl_2_ and dried using anhydrous MgSO_4_. The 4-cyano-*N,N*-diphenylaniline (**1**) was isolated by column chromatography with 61.1% (4.98 g) yield as a yellow powder. ^1^H NMR (CDCl_3_, 500 MHz) d/ppm: 6.94 (d, 2H), 7.20 (d, 4H), 7.25 (d, 2H), 7.39 (t, 4H), 8.05(d, 2H); MS: m/z (EI): 270.12^31^.

*Synthesis of compound **2*** (*4-carboxy-N,N-diphenylaniline*) 4-Cyano-*N,N*-diphenylaniline (1.0 g) and KOH (2.1 g) were firstly dissolved in a mixture of deionized water (30 mL) and glacial acetic acid (20 mL) in a pre-dried three-necked flask and heated at reflux (85 °C) for 48 h. After cooling, hydrochloric acid (1 M) was added dropwise to the reaction solution until the pH value of the reaction solution was adjusted to about 1. Meanwhile, a lot of white precipitate appeared, then it was isolated by filtration and washed with a large amount of deionized water. The obtained white powder of 4-carboxy-*N,N*-diphenylaniline (**2**) was dried in vacuum at 60 °C for 24 h to give an 82% (0.82 g) yield. ^1^H NMR (CDCl_3_, 500 MHz) d/ppm: 7.00 (t, 2H), 7.17 (m, 6H), 7.34 (t, 4H), 7.92 (d, 2H), 10.43 (s, 1H, sharp COOH); MS: m/z (EI): 288.

**Figure 1:**
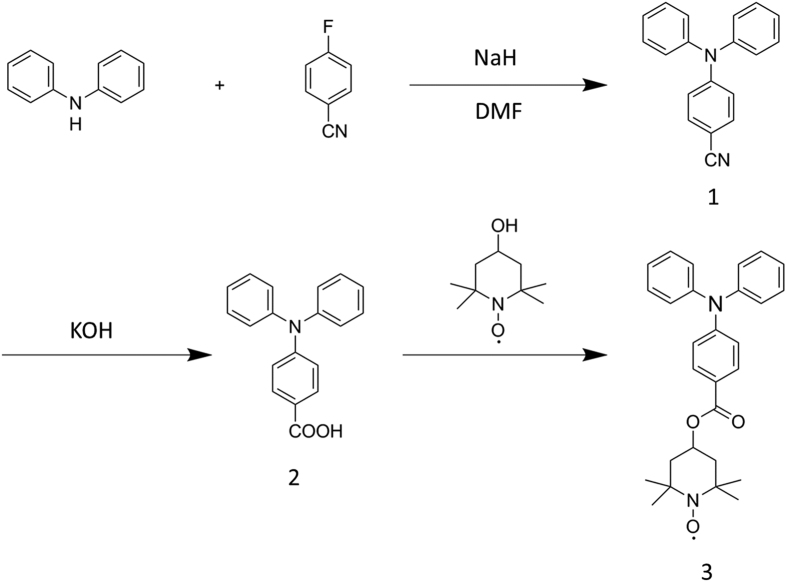
Synthesis route of the TPAT monomer.

*Synthesis of compound **3***(*4-carboxy-N,N-diphenylaniline-2,2,6,6-tetramethylpiperidin-1-yloxy*) 4-Hydroxy-2,2,6,6-tetramethylpiperidine 1-oxyl free radical (1.6 g) and 4-carboxy-*N,N*-diphenylaniline (2.0 g) were dissolved in CH_2_Cl_2_ (50 mL) in a pre-dried three-necked flask, then 1-(3-dimethylaminopropyl)-3-ethylcarbodiimide hydrochloride (0.4 g) as the dehydrating agent and 4-dimethylaminopyridine (1.6 g) as the acylating catalyst were added, and the mixture was stirred for 24 h at room temperature. The reaction mixture was then separated by vacuum filtration. The filtrate was washed with saturated brine three times and the organic phase was dried using anhydrous Na_2_SO_4_. The ester was purified column chromatography using silica gel and petroleum ether/ethyl acetate to afford the title compound (**3**) as a pink powder with 61.8% (1.9 g) yield. MS: m/z (EI): 443. The synthesis of TPAT monomer *via* 1–3 is summarized in Fig. 1.

## Results and Discussion

### Preparation and characterization of the PTPAT film

**Figure 2:**
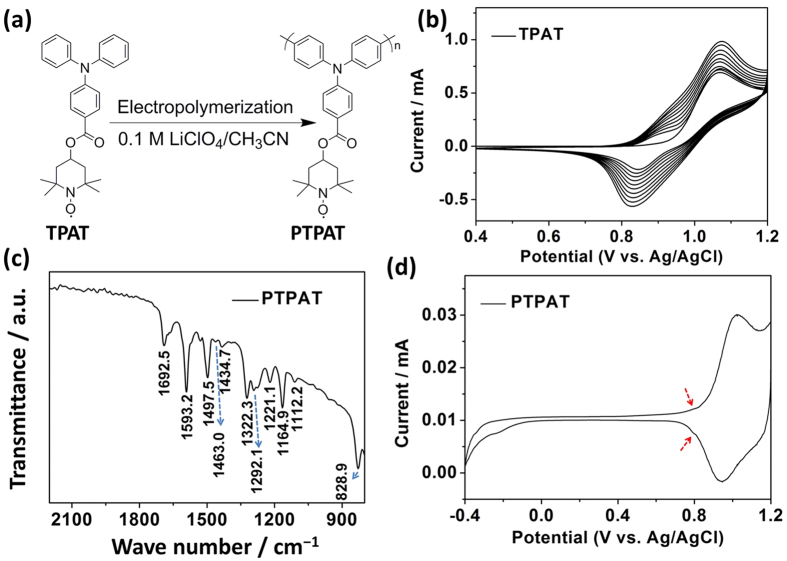
(**a**) Electropolymerization of TPAT; (**b**) Successive CVs of 1 mM TPAT in 0.1 M LiClO_4_/CH_3_CN solution at a scan rate of 100 mV s^−1^; (**c**) FT−IR spectrum of a PTPAT film; (**d**) CV of a PTPAT film in 0.1 M LiClO_4_/CH_3_CN solution at a scan rate of 10 mV s^−1^ (The arrows indicate the presence of weak redox peaks, note Fig. S2).

The PTPAT film was prepared *via* electropolymerization of the TPAT monomer onto the ITO substrate, Fig. 2a. As shown in Fig. 2b, electropolymerization was performed in a solution of 0.1 M LiClO_4_/CH_3_CN containing 1 mM TPAT monomers *via* repetitive cycling at a scan rate of 100 mV s^−1^. In the first cycle, TPAT exhibits a quasi-reversible redox couple with an oxidative peak at 1.07 V (onset at 0.90 V) and a reductive peak at 0.85 V (Fig. S1). During successive CV scans, both the oxidation and reduction currents gradually increased, indicating successful deposition of an electroactive PTPAT polymer film on the ITO surface. In this process, a new oxidation peak appeared at around 0.9 V which is consistent with the behavior of the as-formed PTPAT film on the ITO electrode surface (note below). With an increase in the deposited film thickness, the potential separation between the anodic and cathodic peaks (∆*E* = *E*_p,a_ − *E*_p,c_) increases slightly, consistent with an increase in the internal resistance across the film.

Figure 2c shows the FT−IR spectrum for an as-prepared PTPAT film. The peaks at 1593.2, 1497.5 and 1322.3 cm^−1^ can be ascribed to the fundamental vibrations of the triphenylamine moieties, corresponding to the C=C ring stretching, the C−C stretching and the C−H bending, respectively. The peaks at 1292.1 and 828.9 cm^−1^ are attributed to the C−N stretching of the tertiary amine and the C−H out-of-plane vibration from 1, 4−disubstituted benzene rings. While the stretching of C=O group of ester carbonyl is found at 1692.5 cm^−1^, the peaks at 1112.2, 1164.9 and 1221.1 cm^−1^ are attributable to the stretching of γ_c−o−c_ exsiting in the ester linkage. TEMPO moieties are evidenced by peaks at 1434.7 and 1463.0 cm^−1^ for the C−H stretching of the −CH_3_ and −CH_2_−. The results of FT−IR spectroscopy indicate that TPAT monomers were successfully synthesized and upon electropolymerization, both the triphenylamine and TEMPO moieties were well-retained and incorporated into the PTPAT polymer film^31^.

Figure 2d shows CV of the as-prepared PTPAT recorded in 0.1 M LiClO_4_/CH_3_CN solution at a scan rate of 10 mV s^−1^. The dominant peaks are located at *E*_p,a _= 1.02 and *E*_p,c_ = 0.93 V which can be attributed to the redox reaction of the triphenylamine units of PTPAT. Additional weak redox peaks are observed at 0.80 and 0.78 V arising from the *p*−type doping of nitroxide radical and its reciprocal conversion to the oxoammonium cation (A magnified view of CV in Fig. 2d is shown in Fig. S2). Similarly, another couple of redox peaks appearing at around −0.2 V is related with the *n*−type doping of nitroxide radical, corresponding to the redox process between the aminoxy anions and the nitroxyl radicals^31^. The CV profile is consistent with earlier reports on PTPAT electrochemistry^31^, indicating further the successful fabrication of the PTPAT film.

**Figure 3:**
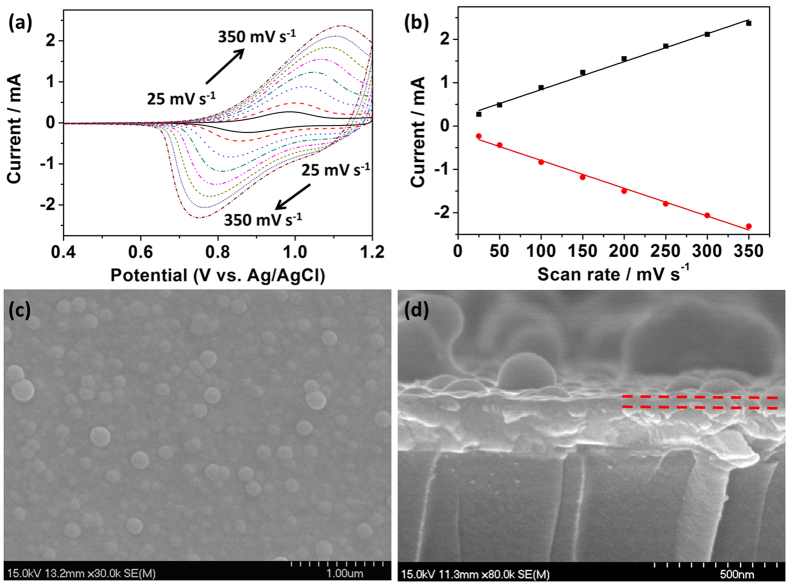
(**a**) CVs of a PTPAT film in 0.1 M LiClO_4_/CH_3_CN solution at different scan rates; (**b**) Plots of the anodic and cathodic peak current densities vs. scan rates; (**c**) Top-down and (**d**) cross-sectional SEM images of the PTPAT film.

Scan rate alternation experiments were conducted to probe the charge transfer within the polymer film. As shown in Fig. 3a, the redox peak of the triphenylamine unit of PTPAT is enhanced with increasing the scan rate within the potential range from 0.4 to 1.2 V. As expected for a surface-bound couple, the anodic and cathodic peak currents are linear with the scan rate (Fig. 3b), indicating that the PTPAT film adheres firmly onto the ITO surface and the charge transfer across the polymer film is not restricted by the diffusion of balance charges (i.e. counterions ClO_4_^−^). Figure 3c,d show the top-down and cross-sectional SEM images of the PTPAT film with images of low magnification shown in Fig. S3. As can be seen from Fig. 3c, the surface of the PTPAT film consists of some spherical nanoparticles with diameters less than 250 nm. The image in Fig. 3d reveals that PTPAT part consists of a thin layer of approximately 50 nm (marked by red dash lines) and globules with diameters in the tens to hundreds of nanometers range grown on the thin layer (a schematic diagram of the PTPAT electrode was illustrated in Fig. S4). The presence of these globules could result in a higher surface contact area between the PTPAT film and the electrolyte solution, which thus favors more rapid doping of counterions.

### Electrochromism performance

**Figure 4:**
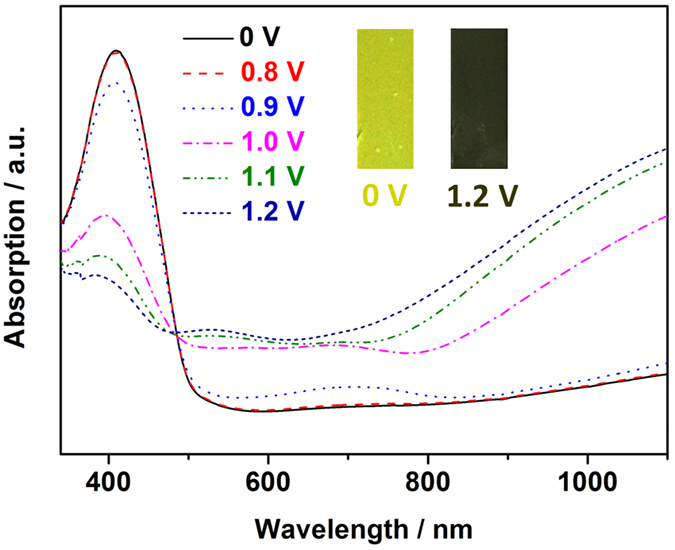
Spectroelectrochemical behavior of a PTPAT film on the ITO glass under different applied potentials in 0.1 M LiClO_4_/CH_3_CN solution (Inset: camera photos of a PTPAT film at 0 V and 1.2 V, respectively).

Figure 4 shows UV-Vis absorption spectra of a PTPAT film under different applied potentials. At an applied potential of 0 V, a well-defined absorption band centered at 409 nm is observed, which can be ascribed to the π–π^*^ transition of the neutral state polymer backbone. With increasing the applied potential, the intensity of this peak decreases, indicating that oxidation of TPA units in the polymer backbone alters the band gap of PTPAT by the doping of counterions. The attenuation in the main band at 409 nm is accompanied with the appearance of charge carrier bands at round 700 and 1100 nm that arises from the evolution of polaron and bipolaron bands^18^,^19^,^20^,^21^,^22^. With increase in applied potentials leading to the change of the film from its neutral state (0 V) to oxidized state (1.2 V), the color of the film turns from yellow to dark green (Fig. 4 inset).

**Figure 5:**
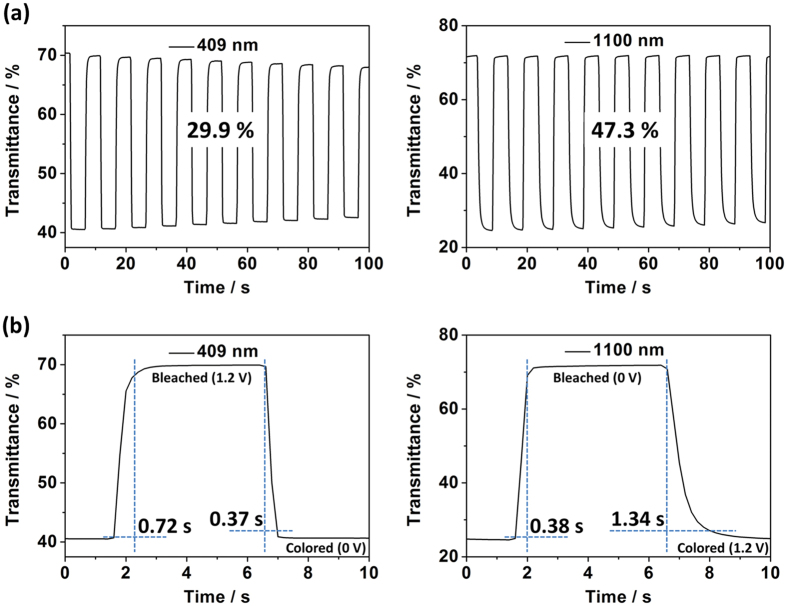
(**a**) Optical contrasts of a PTPAT film monitored at 409 and 1100 nm in 0.1 M LiClO_4_/CH_3_CN solution between 0 and 1.2 V with a residence time of 5 s; (**b**) Electrochromic switching response for a PTPAT film monitored at 409 and 1100 nm in 0.1 M LiClO_4_/CH_3_CN solution between 0 and 1.2 V with a residence time of 5 s.

The electrochromic switching performance of the PTPAT film was examined with a residence time of 5 s at the visible (409 nm) and near-IR light region (1100 nm) between 0 and 1.2 V. Figure 5a displays optical contrasts of the PTPAT film which exhibits a 29.9% contrast at 409 nm and 47.3% at 1100 nm between its neutral and oxidized states. Figure 5b exhibits the switching response of a PTPAT film at 409 and 1100 nm, respectively. The switching time is defined as the time required to reaching 95% of the full change in absorbance after switching the potential. At 409 nm, the switching time is 0.37 s for coloring and 0.72 s for bleaching. At 1100 nm, the switching for bleaching only needs 0.38 s, while the time slightly extends to 1.34 s for coloring. The high optical contrast and rapid switching time make the PTPAT film among the best electrochromic materials based on polytriphenlylamine derivatives^18^,^19^,^20^,^21^,^22^. It is worth to compare, in particular, to the most structurally related polymers, poly(4-cyanotriphenylamine) with pendent -CN or poly(4-nitrotriphenylamine) with pendent -NO_2_^22^. As listed in Table 1, the switching time of PTPAT decreases by almost one order of magnitude as compared to the above two, which confirms the essential role played by the TEMPO moieties. It is also important to note that unsubstituted PTPA cannot be prepared by electropolymerization^16^ and thus TEMPO moieties are also crucial in achieving the good film-forming property of PTPAT in addition to acting as a counterion-reservoir group for rapid switching response.

### Electrochromic mechanism

**Figure 6:**
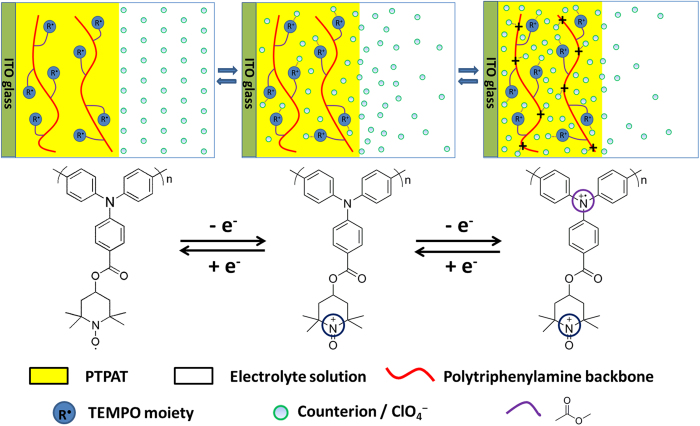
Proposed electrochromic mechanism of the PTPAT film.

It is known that the switching response is dependent on the counterion transport into the polymer layer. An effective way to improve the switching rate is thus to reduce the counterion transporting resistance in the polymer. The usual approach to realize this is to design and prepare nanostructured EC materials that could provide intrinsically high porosity and high surface area by template method^32^,^33^,^34^. However, the report on molecular design to achieve prompt switching response has been rare. Based on our experimental observation, we propose here a new chemical approach to realize rapid switching response by introduction of TEMPO moieties into polyphenylamine backbone. Although TEMPO moieties are not electrochromically active, we speculate that they can function as counterion-reservoir groups to obtain more rapid switching response. A schematic representation of the electrochromic mechanism of the PTPAT film is illustrated in Fig. 6. The electrochromism is presumed to proceed through three steps from neutral state to oxidized state: (1) With the applied potential maintained at 0 V, counterions (ClO_4_^−^) dispersed uniformly in the electrolyte solution; (2) By increasing the potential beyond 0.80 V, the pendent TEMPO moieties at the interface and/or in the interior of the polymer film are oxidized to the oxoammonium cations. Upon oxidation, counterions doping occurs simultaneously to balance the charge. At this stage, electrochromic phenomenon is not observed because TEMPO moiety is an inactive component toward electrochromism. However, it might increase the counterion density near or within the film. (3) By further increasing the potential to 1.02 V, the electroactive triphenylamine units in the skeleton are beginning to oxidize. With the counterions accumulated within or near the PTPAT film, they are ready to balance the oxidized triphenylamine cationic units, thus resulting in rapid color switching. In the reserve process for reduction of the oxidized triphenylamine units, a similar process might occur with the subsequent reduction of the oxidized TEMPO moiety promoting the counterions to move out of the film.

As an additional support for the proposed mechanism, we tested the surface wettability of PTPAT film by measuring the CH_3_CN contact angle (Fig. S5). It was found that the contact angle between CH_3_CN and PTPAT was around 9.1°, which indicates a quite well surface wettability of PTPAT film in CH_3_CN solution. The favorable wettability makes it possible for counterions in the CH_3_CN solution to rapidly dope the PTPAT film, leading to the prompt switching response.

### Stability

**Figure 7:**
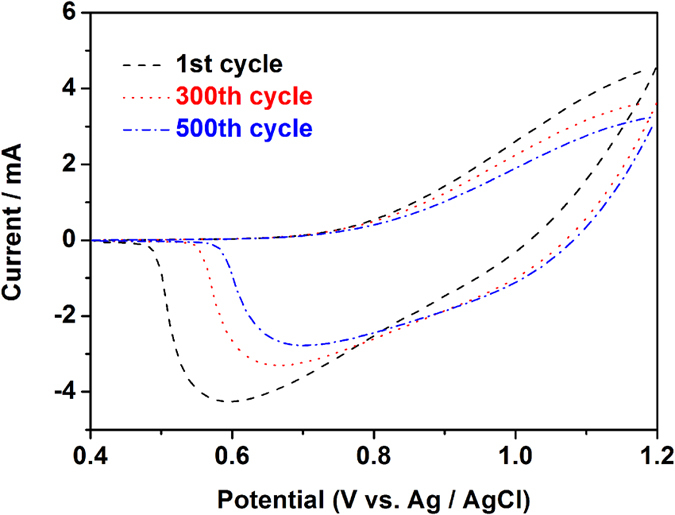
Long-term stability test on the PTPAT film by repeated CV scans in 0.1 M LiClO_4_/CH_3_CN solution between 0.4 and 1.2 V at 500 mV s^−1^.

The electrochemical stability of EC materials during long-term switching between the neutral and oxidized states is one of the most important parameters for application in EC devices. Stability test for the PTPAT film was conducted by successive CV cycles between 0.4 and 1.2 V in 0.1 M LiClO_4_/CH_3_CN solution. As shown in Fig. 7, the PTPAT film remained 86.5% of its original electroactivity after 300 cycles and 74.1% after 500 cycles, which confirms a decent stability of the PTPAT film and provides a promising material candidate for EC device.

## Conclusions

In conclusion, a PTPAT film with TEMPO moieties has been synthesized and firstly applied as an EC material, which shows an excellent film-forming property and electrochromism with reversible color changes between yellow (neutral state) and dark green (oxidized state). The electrochromic performance test on the PTPAT film exhibits a quite high optical contrast of 47.3% at the near-IR region (1100 nm), and a significantly rapid switching time of 0.37 s for coloring and 0.72 s for bleaching in the visible light region (409 nm). The stability of the PTPAT film is also impressive with 74.1% of its original electroactivity retained after 500 repeated cycles. The proposed mechanism suggests that the redox process of pendent TEMPO moieties could greatly improve the electrochromic response of the triphenylamine units with TEMPO serving as a counterion (ClO_4_^−^)-reservoir. The remarkable electrochromic performance of the PTPAT film, especially its blink switching response indicates that this material may serve as a promising candidate for the design and fabrication of an excellent EC device.

## Additional Information

**How to cite this article**: Ji, L. *et al.* A fast electrochromic polymer based on TEMPO substituted polytriphenylamine. *Sci. Rep.* **6**, 30068; doi: 10.1038/srep30068 (2016).

## Supplementary Material

Supplementary Information

## Figures and Tables

**Table 1:**
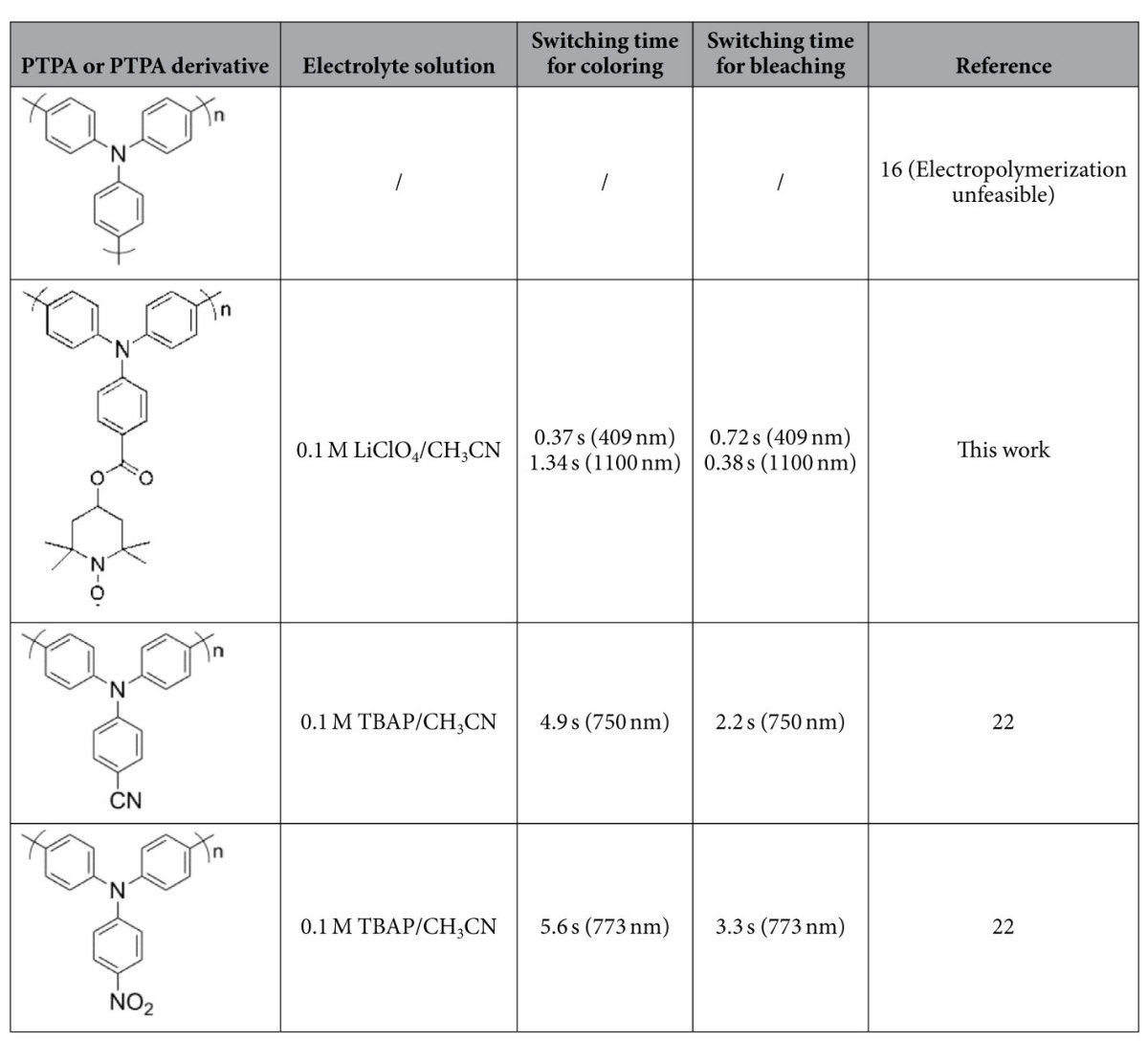


Comparison of the switching time of the PTPAT film and other structurally related polymers with pendent -CN or -NO_2_ (TBAP: tetrabutylammonium perchlorate).
