# Supplementary material for: A fast electrochromic polymer based on TEMPO substituted polytriphenylamine
Source: Sci Rep. 2016 Jul 22;6:30068. doi: 10.1038/srep30068 (PMC4957116; doi:10.1038/srep30068)
Supplement: Supplementary Information [file srep30068-s1.doc]

**Supporting Information**

A fast electrochromic polymer based on TEMPO substituted polytriphenylamine

Lvlv Ji,^ab^ Yuyu Dai,^a^ Shuanma Yan,^a^ Xiaojing Lv,^a^ Chang Su,^c^ Lihuan Xu,^c^ Yaokang Lv,^＊ad^ Mi Ouyang,^a^ Zuofeng Chen^＊b^ and Cheng Zhang^＊a^

*^a^State Key Laboratory Breeding Base of Green Chemistry-Synthesis Technology, College of Chemical Engineer, Zhejiang University of Technology, Hangzhou 310014, China; ^b^Department of Chemistry, Tongji University, Shanghai 200092, China; ^c^College of Chemical Engineering, Shenyang University of Chemical Technology, Shenyang 110142, China; ^d^Department of Chemistry, Tsinghua University, Beijing 100084, China.*


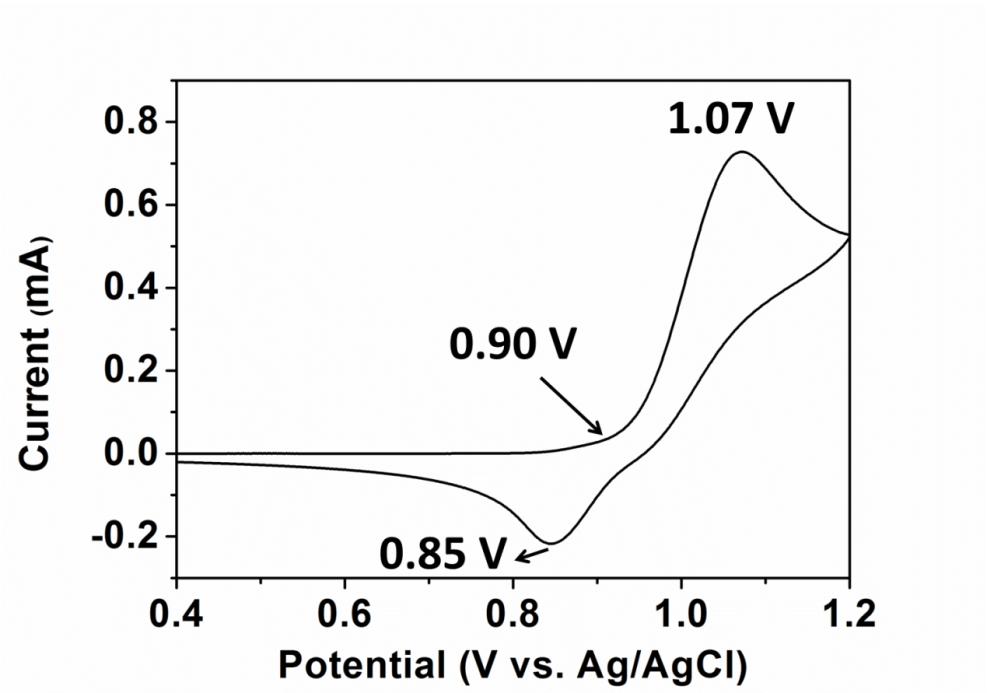


**Fig. S1** The first CV cycle of 1 mM TPAT in 0.1 M LiClO_4_/CH_3_CN solution at a scan rate of 100 mV s^–1^.


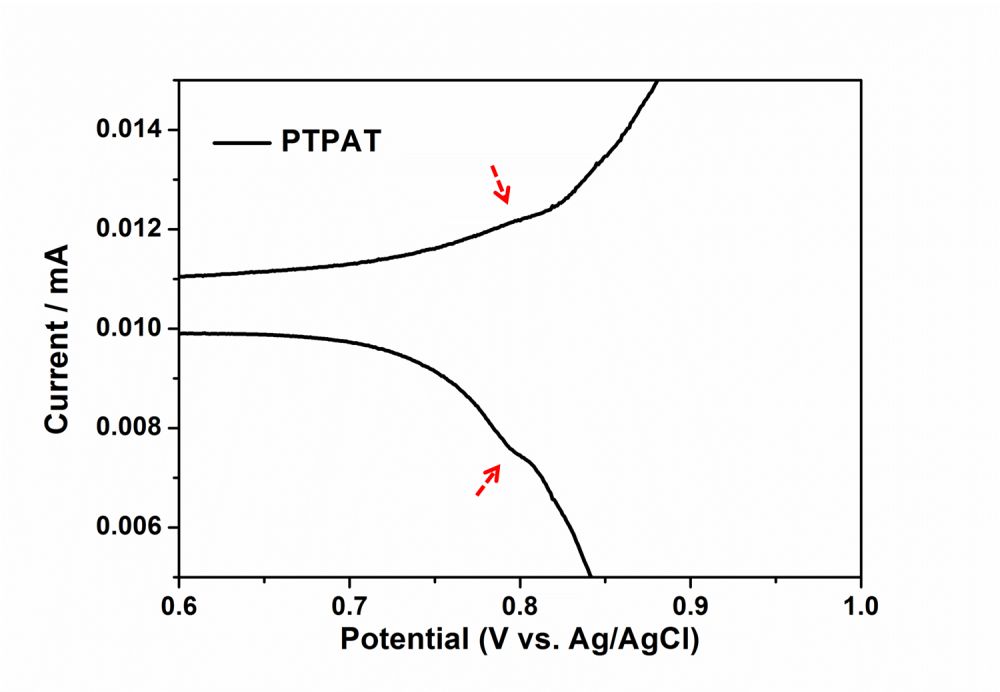


**Fig. S2** A magnified view of CV in Fig. 2d.


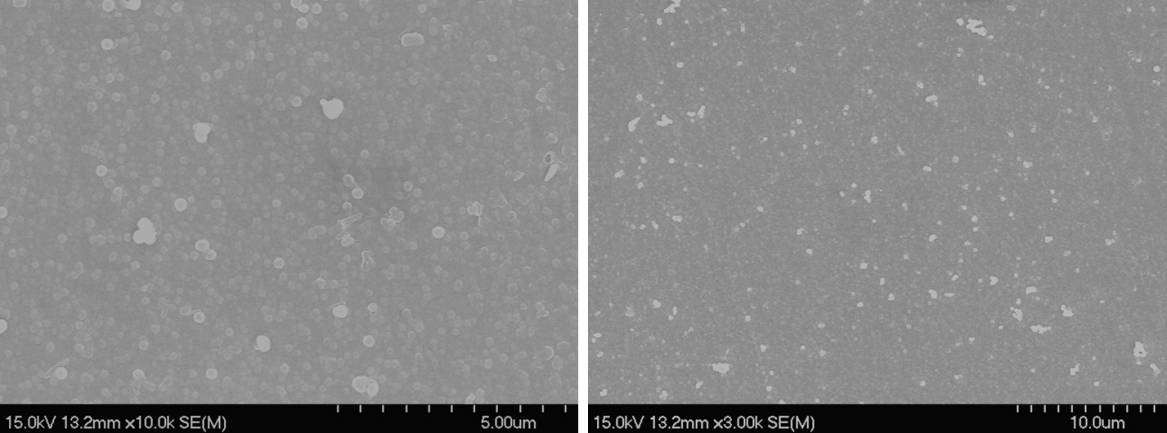


**Fig. S3** Top-down SEM images of the PTPAT film at magnifications of 10 K and 3 K.


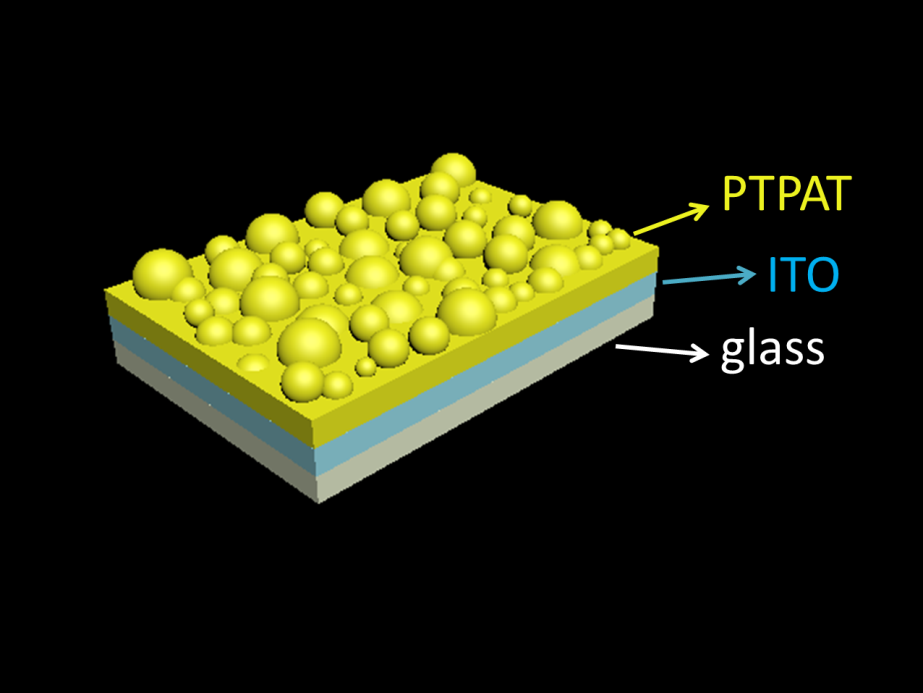


**Fig. S4** Schematic diagram of the PTPAT electrode.


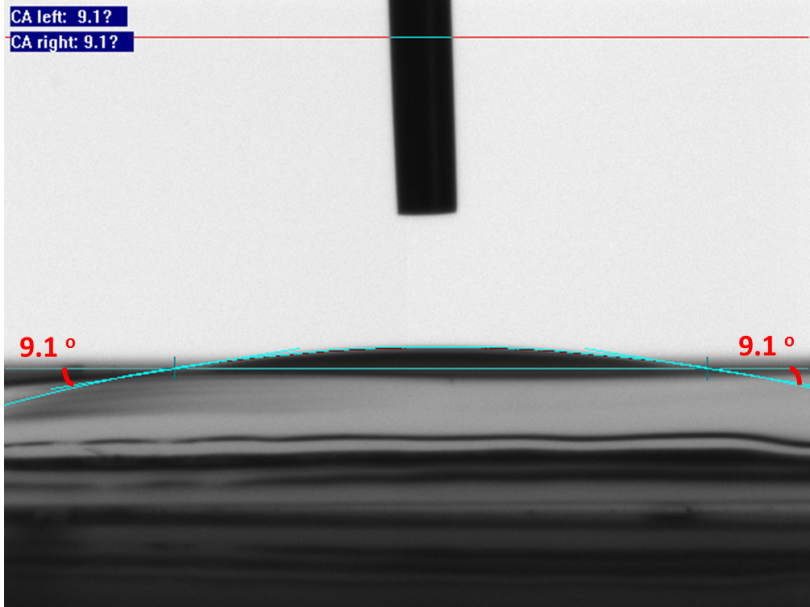


**Fig. S5** Image of CH_3_CN droplet on the surface of the PTPAT film.
